# Predictors of burnout in nurses working in inpatient rooms at a public hospital in Indonesia

**DOI:** 10.11604/pamj.2019.33.148.18872

**Published:** 2019-06-27

**Authors:** Yumi Yestiana, Tri Kurniati, Abdul Aziz Alimul Hidayat

**Affiliations:** 1 Department of Nursing, Rumah Sakit Jantung dan Pembuluh Darah Jantung Harapan Kita Jakarta, Jakarta, Indonesia; 2 Department of Nursing, University of Muhammadiyah Jakarta, Jakarta, Indonesia; 3 Department of Nursing, Faculty of Health Sciences, University of Muhammadiyah Surabaya, Jl Sutorejo No. 59 Surabaya, Indonesia

**Keywords:** Workload, competence, policy, nursing

## Abstract

**Introduction:**

This study aimed to determine the factors that predict the incidence of burnout in nurses who work at the Public Hospital of Tangerang Regency in Banten, Indonesia.

**Methods:**

A cross-sectional design was used in this study. Participants were selected from eight inpatient wards at the Public Hospital of Tangerang Regency (hereinafter termed the hospital) by using a proportionate stratified random sampling method. A total of 133 nurses working in the inpatient wards were recruited. Data were collected using a questionnaire on nursing work schedule setting policy, daily log questionnaire for workload, competency scale and the Maslach Burnout Inventory scale for nurse burnout. Stepwise multiple linear regression was used to analyze the data.

**Results:**

For most respondents (54.1%), the nursing work scheduling policy was appropriate, whereas the average score of nurse workload was 80.42 with SD ± 0.49, and the competency of most nurses was appropriate (64.7%). The average score of nurse burnout was 17.48 with an SD ± 0.50. Work schedule policy and workload were significant burnout predictors, accounting for 87.2% of the variance (Adjusted R^2^=0.872) in burnout among nurses who worked in the hospital's inpatient wards.

**Conclusion:**

Nursing work schedule setting policy and workload were the main factors that led to burnout in nurses working in the inpatient wards. This issue can be overcome by regulating the workload in a balanced manner and applying appropriate policy in the nurse work schedule.

## Introduction

Burnout is a psychological problem and includes emotional and physical fatigue caused by excessive stress that can last a long time. This problem is often found in nurses who work in hospitals in Indonesia. Some studies show that burnout is still a problem for nurses with a high incidence, including Ramdan and Fadly's research in which most of the nurses (56%) working in the AH Samarinda Hospital in East Kalimantan Province, Indonesia, experienced burnout [1]. Likewise, Yumi's study of the incidence of burnout at Tangerang Regency Public Hospital found that 48.0% of nurses suffered from high burnout and the other 52.0% from low burnout. Therefore, managing burnout well is very important to improve nurses' performance in nursing care. Burnout management can be well implemented if various factors that cause burnout are identified, including predictors of burnout incidents in nurses working in inpatient wards at the hospital, such as nursing work schedule policy, nurse workload and competency factors. Several studies have been conducted to determine the factors that predict the incidence of burnout in nurses, such as the Tawale *et al*. research, indicating a correlation between work motivation of nurses and tendency for burnout in which greater motivation is associated with less burnout. Additionally, Indraswari and Ningrum's study revealed a connection between hardiness and burnout in which stronger character was associated with less burnout in nurses. Sari established a relationship between workload, demographic factors (age, marital status), length of service, locus of control, self-esteem and burnout syndrome among working nurses, but the results also disclosed no relation between sex, education level and nurse burnout. Widya kusumastuti and Fauziah concluded that interpersonal communication relates to burnout in nurses, whereas Ramdan and Fadly found a relationship between sex, employment status, workload, support, leadership and nurse burnout but found that age had no correlation with nurse burnout [1].

Among comparable studies in various countries, Lee *et al*. showed that indicators of nursing burnout in Taiwan encompassed age, physical/psychological symptoms, satisfaction, occupational involvement and work environment [2]. Vandenbroeck *et al*. determined that factors for the incidence of nurse burnout in Belgium comprised workload, role conflict, emotional burden and work-home interference [3]. Mudallal, Othmanand and Al Hassan's investigation also uncovered various factors influencing nurse burnout in Jordan, including leadership, working conditions and demographic factors [4]. Li, Ruan and Yuan's study uncovered the relationship of supervisor and coworker support, time off work and work requisition with the incidence of burnout in nurses working in Shanghai hospitals [5]. Some of these studies obtained similar findings such as workload factors, but none pertained to work shift scheduling policy and competencies measured in addition to workload associated with burnout incidents. Both factors are important because several public hospitals in Indonesia have different policies and nurses' competency is still diverse due to the education level of nurses working in public hospitals, ranging from a diploma to bachelor's and master's degrees. Consistent with the work schedule policy factor, workload is a critical element in nurses' psychological working conditions, particularly regarding burnout. The objective of the study was to determine the factors that predict the incidence of burnout in nurses working in inpatient wards of the hospital.

## Methods

A cross-sectional design was applied in this research. A proportionate stratified random sampling technique was used to recruit respondents. Data were collected from 133 nurses working in the hospital who met the following inclusion criteria: served for more than 1 year, not sick or on leave, not the ward's head, willing to participate in this study and working in an inpatient room. Exclusion criteria were as follows: was working in an intensive care room, emergency room or special room and was the ward's head nurse.

Data were collected using a questionnaire with questions on policy, workload, competency and burnout. Data were collected for 6 months at Tangerang District Hospital in Banten, Indonesia. The nurses' work shift scheduling policy was evaluated with a policy questionnaire using a scale to assess its implementation. The policy questions comprised the effectiveness, efficiency, adequacy, equity, responsiveness and accuracy of work schedule arrangements. This instrument consisted of 15 question items with a Likert scale of 1=very unsuitable, 2=unsuitable, 3=appropriate and 4=very appropriate. Cronbach's alpha for the policy variable was 0.83.

Nurse workload was measured using a daily log questionnaire, which was answered by writing the time and type of nursing activities, involving direct care, but not daily activities. The responses were calculated by comparing the percentage of time for implementation of productive activities against the time for nonproductive activities resulting in a high workload when the rate of productive time exceeded optimum productive working time of more than 80% of the nurses' time for activities, an optimum workload when the proportion of productive activity was 80% and when the portion of time for implementation of productive activities was <80% of all the time for activities undertaken by the nurse. Cronbach's alpha for the workload variable was 0.95.

The assessment of nurse competency utilized a competency scale. This instrument consisted of 27 items with a Likert scale of 1=very unsuitable, 2=unsuitable, 3=appropriate and 4=very appropriate. Questions 1-4 asked about professional, ethical and cultural-sensitive practices; 5-24 were related to nursing care and its management; and 22-27 focused on personal and professional quality development. Cronbach's alpha for the nurse competency variable was 0.91.

Burnout was measured by the Maslach Burnout Inventory scale, employing 7 questions on emotional fatigue, 6 concerning depersonalization and 8 regarding decreased achievement. The questionnaire asked respondents to grade their perceived condition using a scale of 0=never, 1=several times a year, 2=once a month, 3=several times a month, 4=once a week, 5=several times a week and 6=daily. Cronbach's alpha for the burnout variable was 0.97. A multiple logistic regression analysis was performed to test the burnout predictors of nurses who worked in the hospital. The level of significance was set at P<0.05.

## Results

Table 1 presents the respondents' characteristics, including age, gender, education, tenure and marital status. A total of 133 respondents were included in the analysis. Most participants (58.0%) were younger than 31 years old, the majority (68%) were females, and their education level was predominantly a nursing diploma (95.0%). The majority of the respondents had worked for less than 5 years (63.0%) and most were married (72.0%). The nursing work shift scheduling policy was split into two categories: appropriate and inappropriate policies. Most deemed the policy appropriate (54.1%). The average workload score of all the participants was 80.42 and the standard deviation was 0.49. Workload score was classified into two levels: level of low workload (≤80%) and high workload (>80%). Nurse competency was organized into two categories: appropriate competency and incompatible competency. Most of the surveyed nurses had appropriate competency (64.7%). The average burnout score of all the respondents was 17.48 with a standard deviation of 0.50. Burnout levels were classified into two categories: high burnout with a score of >18 and low burnout at <18. Table 2 displays the relationship of age, sex, education, employment state, marital status, workload, competency and policy with burnout. The Pearson's correlation test results on those variables revealed that burnout significantly correlated with workload (r=0.885, p <0.01), competency (r=0.712, p <0.01) and policy (r=0.886, p<0.01); however, other variables, namely, age, gender, education, tenure and marital status, had no significant relation to burnout.

**Table 1:** Frequency and percentage of respondent characteristics (n= 133)

Characteristic of Respondent	Frequency	Percentage
**Age**		
>30 years	56	42.0
≤30 years	77	58.0
**Gender**		
Woman	90	68.0
Man	43	32.0
**Education**		
Diploma	126	95.0
Bachelor’s	7	5.0
**Years of service**		
≤5 years	84	63.0
6-10 years	13	10.0
≥11 years	36	27.0
**Marital status**		
Married	96	72.0
Single	37	28.0

**Table 2:** Matrix of the correlations between the independent variables and burnout (n= 133)

Independent Variable	R	P-Value
Age	0.090	0.304
Gender	0.054	0.534
Education	0.043	0.627
Years of service	0.107	0.222
Marital status	0.128	0.143
Workload	0.885	0.000
Competency	0.712	0.000
Work shift scheduling policy	0.886	0.000

A multiple linear regression analysis was conducted to test the variables that significantly indicated burnout. After examining the assumption of multiple linear regression of independent variables, including age, sex, education, tenure, marital status, workload, competency and policy, only two variables were found to be significant predictors of burnout in nurses working in the inpatient wards of the hospital. Table 3 shows that workload and work scheduling policy influenced 87.2% (Adjusted R^2^=0.872) of burnout incidents. The strongest predictor of burnout was the work schedule policy (β=0.499), followed by the workload (β= 0.494). The work schedule policy affected the burnout score with B=0.500 (p<0.01). This variable projected that the probability of a nurse experiencing burnout would be 0.500 times higher if the work schedule policy was not well managed, as much as workload with B=0.500 (p<0.01), suggesting that a nurse's chance of experiencing burnout would predictably be 0.500 times greater if the workload was not properly managed. If the workload score increased by 1, the burnout score went up by 0.500 and if the work scheduling policy score increased by 1, the burnout score would also rise by 0.500.

**Table 3:** Nurse burnout predicting factors (n= 133)

Predictor	B	SE	Beta	t	P
Workload	0.500	0.058	0.494	8.629	0.000
Work shift scheduling policy	0.500	0.067	0.499	7.413	0.000
Constant	23.500				
R= 0.938; Adjusted R^2^= 0.872					

## Discussion

This study identified the importance of factors affecting the occurrence of burnout incidents in nurses working in the inpatient wards of the hospital. The results revealed that work shift schedule policy and workload collectively accounted for 87.2% of burnout incidents. Work shift scheduling policy was the strongest indicator of burnout incidence in nurses serving in the inpatient wards at the hospital. The policy in shift arrangement of the work schedule may at times impact nurses' working conditions and their circadian rhythm, in addition to not being compensated by a good quality break during working hours, as nurses must remain at the ready and standby even during breaks and off-hours. Conversely, if the work shift schedule adjustment policy improves the nurses' satisfaction, burnout incidents could be averted [6].

Fatigue is a functional reaction from the center of consciousness, namely, the cerebral cortex, which is influenced by two antagonistic systems, namely, the inhibitory system (inhibition) and the driving system (activation) [7]. The inhibitory system is in the thalamus and can reduce human ability to act and cause a tendency to sleep. According to Nurmianto, the occurrence of fatigue is due to static muscle loading so that blood flow to the muscle decreases, which results in lactic acid accumulation. In addition, it is also due to muscle load that is not evenly distributed in particular tissues. If in a long period of time someone constantly has to do the same motion, the blood circulation becomes disrupted, and the person becomes tired quickly. The policy elements of nurses' service that cause burnout events include the duration of work, type of work shift and rest time. The length of time a person works each day is generally 6-8 hours, and the remaining hours are for rest or life with the family and community. The labor law in Indonesia states that each institution is obliged to follow the provisions of work time for workers who are employed as follows: 7 hours a day and 40 hours a week for 6 working days in 1 week or 8 hours a day and 40 hours a week for 5 working days in a week.

A consequence of working with a shift system is that it often involves long working hours or long work shifts. Kontz [8] recommends that working hours should not exceed 12 hours a day or 55 hours a week. The results show that workers who work more than the recommended work time in a day but with less than the maximum working time for the week are preferred because they have many advantages such as more time spent with the family, fewer consecutive night shifts and longer holidays. However, the impact caused in terms of safety is that it can increase the number of accidents, reduce work performance and increase errors. Studies that have been conducted using subjective or objective measurements of fatigue generally support that there is a relationship between long working hours and the incidence of fatigue. This association can be seen in the research in Japan by Levy [9] who reported that the number of weekly working hours of workers in Japan increased by ¼ from male workers with working hours >60 hours per week. Other studies have shown that judging from long working hours, there is an association between fatigue and health problems such as karoshi-"dead due to long working hours". Karoshi is a syndrome of heart attacks such as stroke and myocardial infarction.

The type of work shift consists of four main types of working hours. They include daily work, which is the work period between 7am and 6pm, and permanent shift, which is a work system in which routine work schedules are followed or are the same every day. Permanent shift is divided into 3 types, namely, morning shift (6am - 2pm), afternoon shift (2pm - 10pm) and night shift (10pm - 6am). Rotating shift is a work system in which there is variation between several shifts. The 2-shift system (morning and evening or evening and night) is usually used, although there are also those who use a 3-shift rotation system (including all three). Roster work or flexible rotating shift is another type of work shift. In arranging work shifts, things that need to be considered include the length of work in one shift, many shifts done before rest days, many rest days in one week, whether there is an increase in working hours or overtime, the length of rest used between shifts, length of rest during the shift, and whether the work schedule is regular and predictable. Work shift settings can affect the incidence of fatigue or stress. Workers with morning shifts and night shifts often feel sleepy and tired during shifts. This effect is because their circadian rhythm is disturbed. Night shift workers are forced to sleep during the day even though the circadian rhythm forces workers to stay awake during the day. Therefore, sleep time is shorter. Additionally, morning shift workers also have short sleep time because they are forced to leave early to go to work, which can make them tired quickly during work [8].

Kontz [8] states that one of the main reasons a person experiences fatigue is insufficient rest. This factor can result from working at the wrong time (shift work) or long working hours. Work requires the deployment of energy and the use of body organs in a coordinated manner. By its nature, work is anabolic, which is the breakdown or use of parts of the body to function. In fatigue, the nervous system is primarily functioning using its sympathetic component. Such a situation cannot occur continuously, and a break that gives the body the opportunity to recover is needed. Then, the rest is catabolic, which allows recovery in the body. In the labor law, rest periods include the following: rest between working hours, at least half an hour after working for 4 hours continuously, and the rest period does not include working hours and weekly breaks at least once a day for 6 working days in a week or 2 days for 5 working days in a week. Annual break, at least 12 working days for 6 working days in a week or 10 working days for 5 working days in a week, after the worker has been employed for 12 (twelve) months continuously; and rest properly to perform the obligations/fulfill worship according to his/her religion. Establishing a good rest time especially for heavy work reduces the occurrence of illness and absenteeism. Experience shows that short breaks are often better than a long break. Research shows that establishing the appropriate rest time has a positive impact on productivity.

This study's findings are consistent with the Winwood *et al*. study, which examined the relationship between work fatigue and division of work shifts. They found out that the more shift work the workers do, the more severe the level of fatigue they experience [10]. The authors observed the frequency of shift work as an independent variable associated with work fatigue, including both physical and mental aspects; it is a long phase of energy deficiency that can lead to a decrease in workers' functional capacity [11]. Workload was distinguished as the second strongest factor in predicting the incidence of burnout among nurses working in the inpatient wards of the hospital. Nursing workload comprises all activities performed by a nurse on duty in a unit of nursing service. Workload could be either quantitative or qualitative. Quantitative workload is typically the number of tasks that must be done to meet patients' health needs, whereas qualitative load is the level of responsibility for providing care to patients. The nurse's workload standard should always be consistent with nursing care oriented to patient's needs. To produce effective and efficient services, nurses' availability should be optimally matched with the existing workload. Nurses' workload is influenced by the constantly changing patient conditions, average number of hours of care needed to provide immediate service to the patient and number of additional tasks to be performed by the nurse during working time [12].

Another investigation conducted by Kiekkas *et al*. demonstrated that burnout syndrome had a significant relationship with the nurse's workload (p value= 0.005). Kiekkas *et al*. also mentioned that a high workload specifically affects one of the dimensions of burnout syndrome, namely, physical and emotional exhaustion [13]. According to Belenky, an overly heavy workload can make a worker suffer from disorders or illness due to work. Excessive workload may prompt physical or mental fatigue and emotional reactions such as headaches, indigestion and irritability [14, 15]. Similarly, the work environment also contributes to 73% of burnout [16]. In contrast, too little workload where repetition of motion occurs can lead to boredom and a sense of monotony. Tedium in regular daily work caused by too few tasks or jobs yields a lack of attention to work, potentially endangering workers [17].

In general, the relationship between workload and work capacity is influenced by a variety of factors that are very complex, both internal and external, including tasks performed by a nurse, work organization, which includes the length of time worked, rest time, work shift and the employment system. The work environment can provide additional burdens. Workload involves internal factors such as: somatic factors (gender, age, body size, nutritional status, health conditions, etc.) and psychological factors (motivation, perception, trust, desire, satisfaction, etc.) [18]. Workload can be distinguished quantitatively and qualitatively. Quantitative workload is a person who works in large quantities in accordance with the time given, and qualitative workload is someone who works with tasks that are repetitive, of various types and have challenges [19].

## Conclusion

This study aimed to determine the factors that predicted the burnout of nurses who work in the inpatient rooms at the Public Hospital of Tangerang Regency in West Java, Indonesia. The examined factors were age, gender, education, tenure, marital status, workload, competency and work scheduling policy. The results showed that workload and work scheduling policy are related to burnout among nurses working in the inpatient rooms. However, the most significant predictor of burnout incidents was the work schedule policy, followed by workload. These variables accounted for 87.2% of the variance in burnout incidents in nurses working in the inpatient wards of the hospital.

### What is known about this topic

- Incidence burnout is an indicator to manage workload and work shift scheduling policy in the hospital;
- The strategy of hospitalization to improve burnout is to manage workload and work shift scheduling policy;
- Evidence shows that the workload and work shift scheduling policy is predictors of burnout.

### What this study adds

- The presence of burnout in nurses as a whole in the study area shows the importance of health for nurses;
- The presence of variations in the incidence of burnout in nurses in hospitals shows the magnitude of the challenge of the control programmes;
- Workload and work shift scheduling policy fluctuations can affect the incidence of burnout for nurses.

## Competing interests

The authors declare no competing interests.

## References

[1] Ramdan IM, Fadly ON (2016). Factor Analysis Associated with Burnout in Mental Health Nurses. JKP.

[2] Lee HF, Yen M, Fetzer S, Chien TW (2015). Predictors of burnout among nurses in Taiwan. Community Ment Health J.

[3] Vandenbroeck S, Van Gerven E, De Witte H, Vanhaecht K, Godderis L (2017). Burnout in Belgian physicians and nurses. Occupational Medicine.

[4] Mudallal RH, Othman WaM, Al Hassan NF (2017). Nurses' Burnout: the influence of leader empowering behaviors, work conditions, and demographic traits. Inquiry.

[5] Li L, Ruan H, Yuan WJ (2015). The relationship between social support and burnout among ICU nurses in Shanghai: a cross-sectional study. Chinese Nursing Research.

[6] Nwafor CE, Immanel EU, Obi-Nwosu H (2015). Does nurses' self-concept mediate the relationship between job satisfaction and burnout among Nigerian nurses. International Journal of Africa Nursing Sciences.

[7] Peretie MM, Samory E (2018). Occupational burnout, understanding and acting. Soins Gerontologie.

[8] Konz S (2000). Work/rest: Part I-Guidelines for the practitioner. Ergonomics Guidelines and Problem Solving.

[9] Levy BS (2006). Occupational and environmental health: recognizing and preventing disease and injury.

[10] Winwood PC, Winefield AH, Lushington K (2006). Work-related fatigue and recovery: the contribution of age, domestic responsibilities and shiftwork. J Adv Nurs.

[11] Dorrian J, Lamond N, van den Heuvel C, Pincombe J, Rogers AE, Dawson D (2006). A pilot study of the safety implications of Australian nurses' sleep and work hours. Chronobiol Int.

[12] Ramli H, Tamsah H (2016). The Effect of Dual Role Conflict, Work and Fatigue Expenses (Burnout) with Female Nurse Performance at I Lagaligo Hospital, East Luwu Regency. Jurnal Mirai Management.

[13] Kiekkas P, Spyratos F, Lampa E, Aretha D, Sakellaropoulos GC (2010). Level and correlates of burnout among orthopaedic nurses in Greece. Orthopedic nursing.

[14] Belenky G, Wu LJ, Jackson ML (2011). Occupational sleep medicine: practice and promise. Progress in brain research.

[15] Hariyono W, Suryani D, Wulandari Y (2012). The relationship between workload, work stress and the level of conflict with nurses' work fatigue in Yogyakarta Islamic Hospital PDHI, Yogyakarta City. Jurnal Kesehatan Masyarakat (Journal of Public Health).

[16] Hu SX, Luk AL, Smith GD (2015). The effects of hazardous working conditions on burnout in Macau nurses. International Journal of Nursing Sciences.

[17] Caruso CC (2014). Negative impacts of shiftwork and long work hours. Rehabilitation nursing: the official journal of the Association of Rehabilitation Nurses.

[18] Adarkwah CC, Schwaffertz A, Labenz J, Becker A, Hirsch O (2018). Burnout and work satisfaction in general practitioners practicing in rural areas: results from the HaMEdSi study. Psychol Res Behav Manag.

[19] Watson AG, McCoy JV, Mathew J, Gundersen DA, Eisenstein RM (2019). Impact of physician workload on burnout in the emergency department. Psychol Health Med.

